# Docking Studies and Biological Activity of Fosinopril Analogs

**DOI:** 10.1155/2014/721834

**Published:** 2014-07-06

**Authors:** Jayant Choudary, Suvarna G. Kini, Sreedhara Ranganath Pai Karkala, Muhammad Mubeen

**Affiliations:** ^1^Department of Chemistry, Manipal College of Pharmaceutical Sciences, Manipal University, Manipal 576104, India; ^2^Department of Pharmacology, Manipal College of Pharmaceutical Sciences, Manipal University, Manipal 576104, India

## Abstract

The purpose of the present study was to determine the angiotensin-I converting enzyme inhibitory activity of few novel Fosinopril derivatives which were predicted to possess better ACE inhibitory activity and lesser side effects than the existing drug molecule. *In vitro* study was carried out to determine ACE inhibitory activity of six different Fosinopril analogs by spectrophotometric assay procedure. Analog A2 showed the highest activity compared to other analogs and as well as Fosinopril itself. Docking studies of the compounds were done with the help of VLife MDS 3.0 software using GRIP batch docking method to find out which derivative had a better docking with ACE. The compounds which showed the highest negative score in docking have also exhibited good ACE inhibitory activity.

## 1. Introduction

Angiotensin converting enzyme (ACE, EC 3.4.15.1) inhibitors work by inhibiting the action of angiotensin converting enzymes, thus preventing the conversion of angiotensin to angiotensin-II. ACE inhibitors are widely used in the treatment of hypertension as well as heart failure [1, 2]. Fosinopril is the first and only phosphonate containing ACE inhibitor. The search for ACE inhibitors that lacked the sulfhydryl group leads to the investigation of phosphorus containing compounds. The phosphinic acid is capable of binding to ACE in a manner similar to enalapril. The interaction of the zinc atom with the phosphinic acid is similar to that seen with sulfhydryl groups [3, 4].

**Scheme 1:**
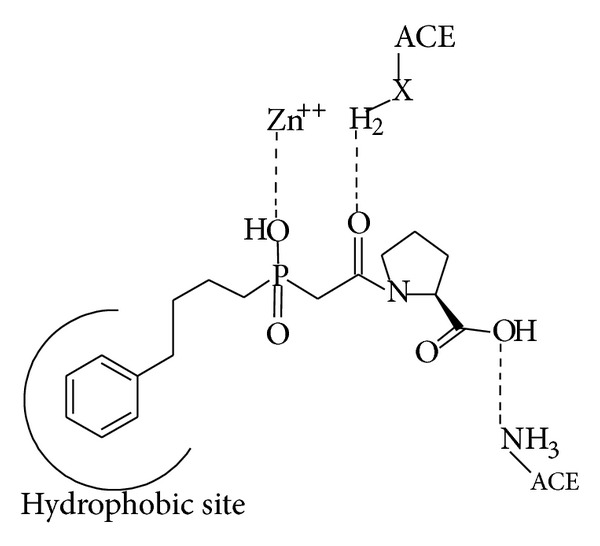
Interaction of Fosinopril with ACE [4].

Additionally, this compound is capable of forming the ionic, hydrogen, and hydrophobic bonds. A feature unique to this compound is the ability of the phosphinic acid to more truly mimic the ionized, tetrahedral intermediate of peptide hydrolysis. Structural modifications to investigate more hydrophobic, C-terminal ring systems, lead to a 4-cyclohexylproline analog of the original phosphinic acid. This compound, Fosinoprilat, was more potent than captopril but less potent than enalaprilat. Fosinoprilat proved to have the same problem as enalaprilat and other carboxylate-containing ACE inhibitors like poor bioavailability. The solution fortunately was very similar—the addition of a hydrophobic side chain to modulate the ionization characteristics of the molecule—thus Fosinopril was developed. Fosinopril is administered as a prodrug and is converted* in vivo* to the active form Fosinoprilat (Scheme 1).

## 2. Results and Discussion

### 2.1. Chemistry

Fosinopril containing analogs of* trans*-4-phenyl-L-proline (A1), 1,2,3,4-tetrahydro isoquinoline-3-carboxylic acid (A2),* trans*-4-cyclohexyl-L-proline (A3),* trans*-4-cyclohexyl-L-proline benzyl ester (A4), and L-proline (A5 and A6) were used for this particular study. These six Fosinopril analogs were given as gift samples by Ranbaxy Lab. Ltd.

### 2.2. *In Vitro *Angiotensin-I Converting Enzyme Inhibitory Activity

This method is based on the cleavage of the substrate hippuryl-glycyl-glycine by ACE and subsequent reaction with trinitrobenzenesulfonic acid to form 2,4,6-trinitrophenyl-glycyl-glycine [5]. The method relies on spectrophotometric determination of hippuric acid. It involves a colorimetric reaction of hippuric acid with benzene sulfonyl chloride [6].

**Figure 1:**
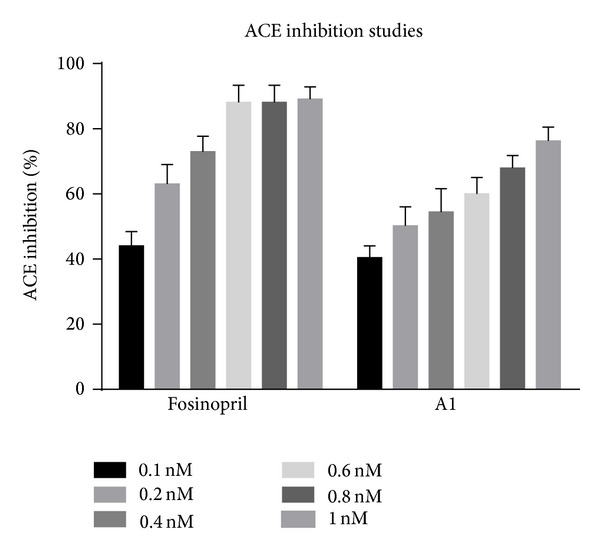
Percent ACE inhibition by Fosinopril and A1.

**Figure 2:**
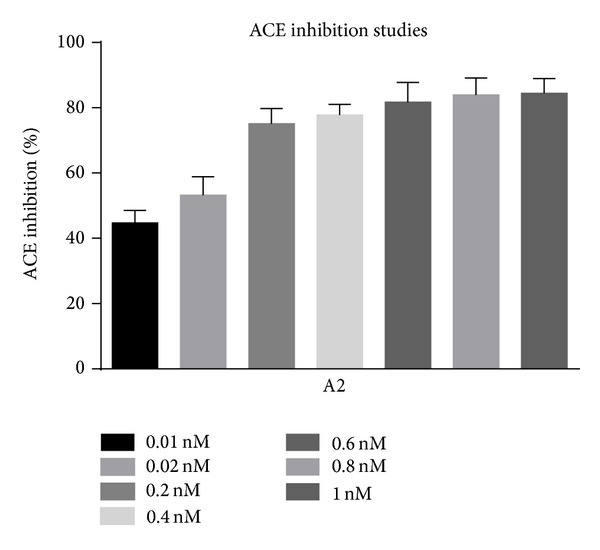
Percent ACE inhibition by A2.

**Figure 3:**
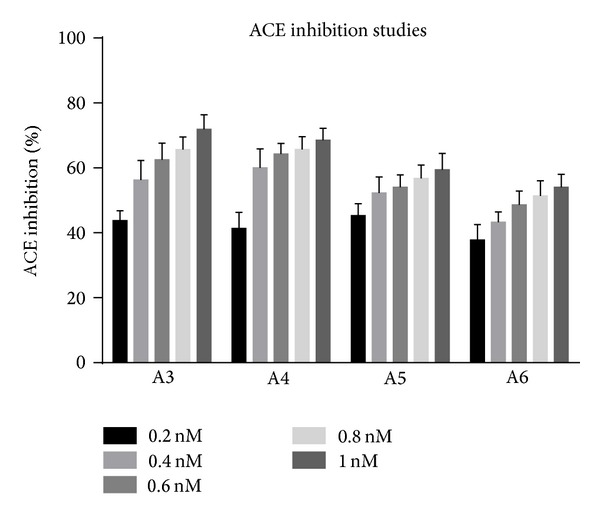
Percent ACE inhibition by A3, A4, A5, and A6.

**Figure 4:**
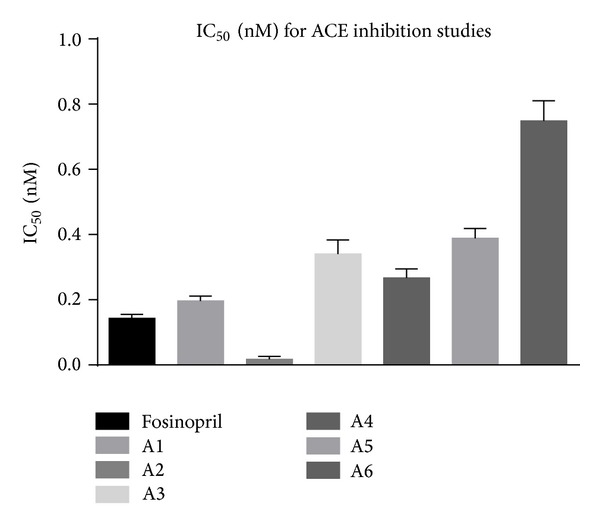
Graph of IC_50_ values of analogs.

The assay system consisted of 200 *μ*L of substrate solution, water, and drug solution in a volume of 0.25 mL. The reaction was started by the addition of 40 *μ*L rat lung extract. After 30 minutes of incubation at 37°C, the reaction was stopped by adding 2 mL of stop solution (1.195 g HEPES and 0.0465 g of EDTA were dissolved in 40 mL distilled water. The pH was adjusted to 9.0 with 1 mol/lit sodium hydroxide and diluted to 50 mL with distilled water). Then 0.5 mL distilled water and 1.0 mL colour reagent were added, mixed vigorously, left for 5 min, and centrifuged at 3000–4000 rpm for 10 minutes at room temperature to remove any precipitate. The corresponding enzyme inhibitors blanks were assayed in the same manner except for adding the stop solution before the lung extract. The absorbance of the yellow product of the reaction between liberated hippurate and cyanuric chloride was measured spectrophotometrically at 405 nm against distilled water as blank [7].Controls without drug were run simultaneously in all cases. The results of this study are given in Tables 1, 2, and 3 and Figures 1, 2, and 3 and the IC_50_ values are given in Table 4 and Figure 4. “One inhibitory unit is the amount of inhibitor that suppressed the ACE activity by one unit.”

### 2.3. Docking Simulation

**Figure 5:**
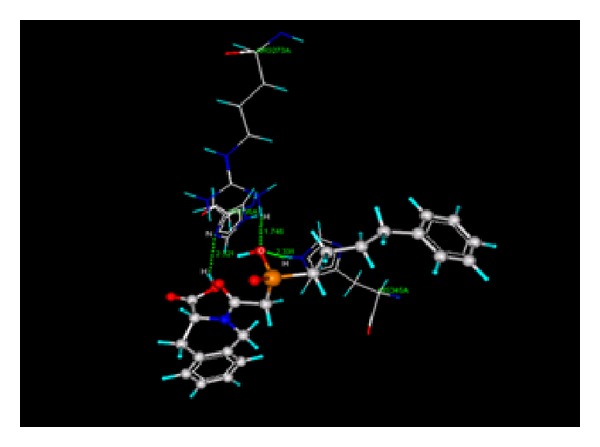
Hydrogen bond of A2 (green dotted lines).

**Figure 6:**
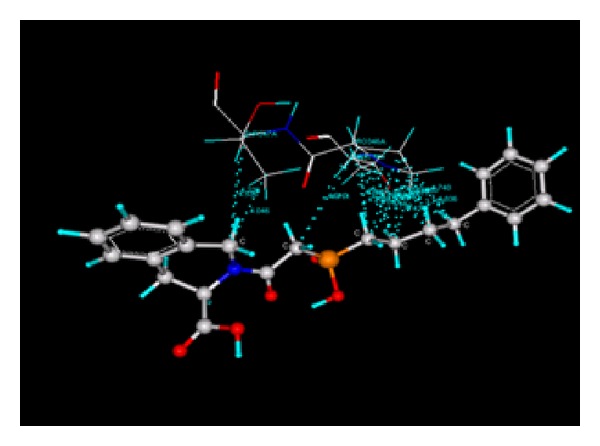
Hydrophobic bond of A2 (blue dotted lines).

**Figure 7:**
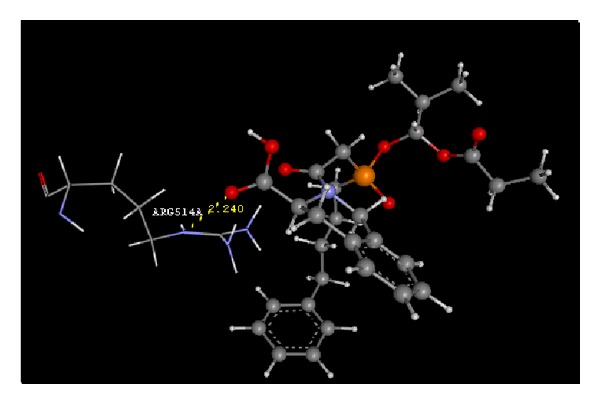
Charge interaction of A2 (yellow dotted lines).

**Figure 8:**
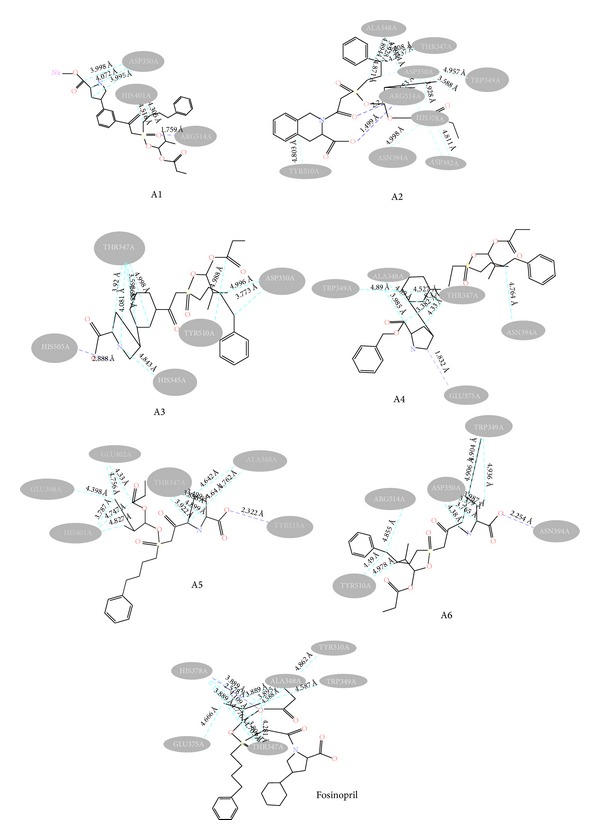
Ligplot of six analogs along with Fosinopril showing hydrogen bond indicated by dark dotted lines and hydrophobic bonds by light dotted lines with respective amino acid residues of the ACE.

Docking study was done by GRIP batch docking method with the help of VLife MDS 3.0 software. The crystal structure of ACE in complex with an irreversible inhibitor (PDB ID: 1R4L) for antihypertensive docking studies was obtained from the protein data bank [8]. The parameter fixed for docking simulation was like number of placements: 30, rotation angle: 30°, and exhaustive method. The ligand forming most stable drug-receptor complex is the one which is having minimum dock score. After docking simulation, the best docked conformer of each ligand was checked for various interactions with receptor like hydrogen bonding, hydrophobic bonding, van der Waals, and charge interaction. Compound A2 was found with highest negative dock score (Table 5), meaning that it forms most stable drug-receptor complex amongst all the compounds. It was found to form hydrogen bonding (Figure 5), hydrophobic bonding (Figure 6), and van der Waals interaction. Amongst all the analogs, A3 was not found to exhibit hydrogen bonding with the receptor through “O” of −OH of Phosphoramidon moiety. Analogs A1, A2, A4, A5, A6, and Fosinopril form hydrogen bonding with residues Arg273 and Thr371 by “O” atom of −COOH group of proline moiety and “O” −OH group of phosphoramidon moiety. All the analogs were found to have hydrophobic bonding. Some of the residues involved in this type of interaction are Leu144, Asp269, Met270, Pro346, Thr347, His345, Ala348, Thr360, Leu370, and Thr445. Thr347 and Thr371 were common residues for hydrophobic bonding of all analogs including Fosinopril. All the analogs including Fosinopril were found to show van der Waals' interaction with common residues Arg273, Thr347, Thr371, Tyr510, and Tyr515. Only A2 was found to show charge interaction (Figure 7) by interaction of “O” atom of −COOH group of tetrahydroisoquinoline-3-carboxylic acid with three “N” atoms of Arg514. The ligand plot of all analogs and Fosinopril is shown in Figure 8 using VLife MDS 3.0.

## 3. Conclusion

*In vitro *studies revealed that the analog A2 exhibited highest ACE inhibition activity than other analogs including Fosinopril. The percent inhibition of ACE activity of different analogs is as follows: analog A2 showed highest activity followed by Fosinopril, A1, A4, A3, A5, and A6, respectively. In docking study analog A2 was found to have minimum dock score indicating highest binding with the receptor and* in vitro* studies also showed the same analog having highest ACE inhibitory activity. Hence we can conclude that analog A2 can be considered as a better molecule than Fosinopril and should be investigated further.

## Figures and Tables

**Table 1:** Percent inhibition of ACE by Fosinopril and A1 at different concentrations.

Analog	Percent inhibition					
	0.1 nM	0.2 nM	0.4 nM	0.6 nM	0.8 nM	1 nM
Fosinopril	44.07 ± 4.35	63.03 ± 6.01	68.72 ± 3.99	72.98 ± 4.76	88.15 ± 5.20	89.09 ± 3.82
A1	40.45 ± 3.60	50.20 ± 5.83	54.46 ± 7.14	60.04 ± 4.99	67.97 ± 3.84	76.25 ± 4.25

**Table 2:** Percent inhibition of ACE by A2 at different concentrations.

Analog	Percent inhibition						
	0.01 nM	0.02 nM	0.2 nM	0.4 nM	0.6 nM	0.8 nM	1 nM
A2	44.72 ± 3.85	53.17 ± 5.73	75.12 ± 4.66	77.80 ± 3.21	81.74 ± 6.09	83.98 ± 5.11	84.45 ± 4.52

**Table 3:** Percent ACE inhibition by A3, A4, A5, and A6 at different concentrations.

Analog	Percent inhibition				
	0.2 nM	0.4 nM	0.6 nM	0.8 nM	1 nM
A3	43.75 ± 3.05	56.26 ± 5.43	62.50 ± 5.11	65.62 ± 3.96	71.87 ± 4.50
A4	41.41 ± 4.88	60.05 ± 5.24	64.27 ± 3.25	65.70 ± 3.89	68.53 ± 3.67
A5	45.29 ± 3.65	52.30 ± 4.91	54.05 ± 3.79	56.77 ± 4.14	59.43 ± 5.02
A6	37.80 ± 4.76	43.29 ± 3.15	48.64 ± 4.20	51.35 ± 4.73	54.03 ± 3.99

**Table 4:** IC_50_ values of analogs.

Analog	IC_50_ (nM)
Fosinopril	0.143 ± 0.012
A1	0.196 ± 0.015
A2	0.017 ± 0.009
A3	0.340 ± 0.044
A4	0.267 ± 0.028
A5	0.389 ± 0.030
A6	0.749 ± 0.061

**Table 5:** Docking scores of the Fosinopril analogs.

Analog	Dock score
A1	−78.9549
A2	−81.0598
A3	−72.0179
A4	−76.0455
A5	−68.0202
A6	−58.0563
Fosinopril	−79.0399
